# Supplementary figures and images for: Research on 3D point cloud alignment algorithm based on SHOT features
Source: PLoS One. 2024 Mar 27;19(3):e0296704. doi: 10.1371/journal.pone.0296704 (PMC10971591; doi:10.1371/journal.pone.0296704)

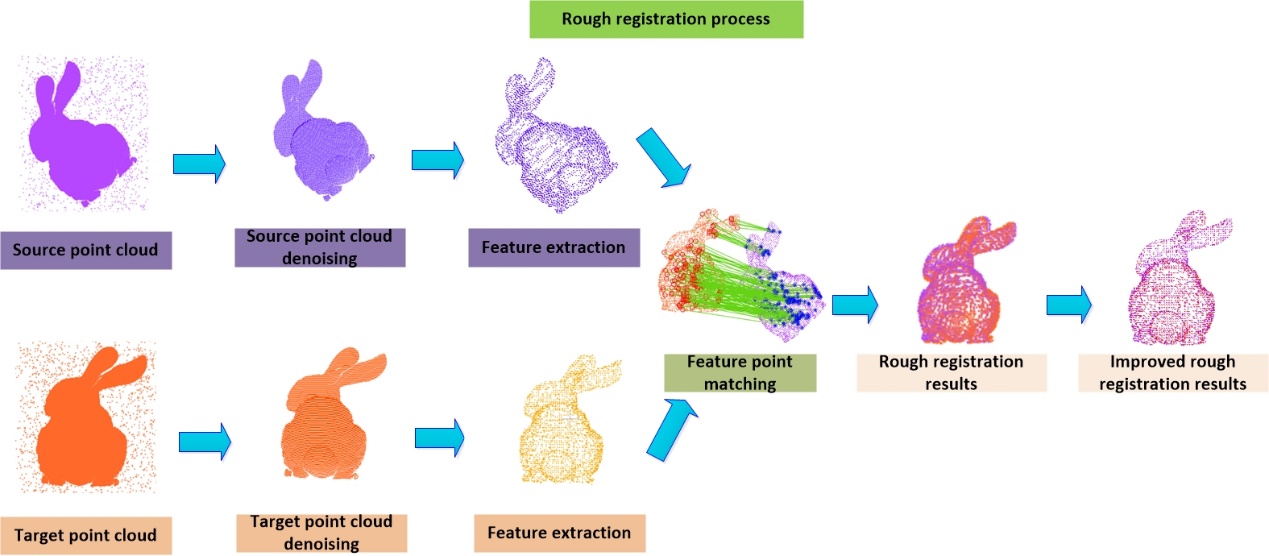


Fig 1


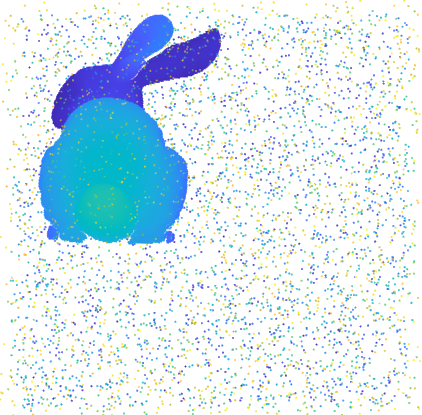

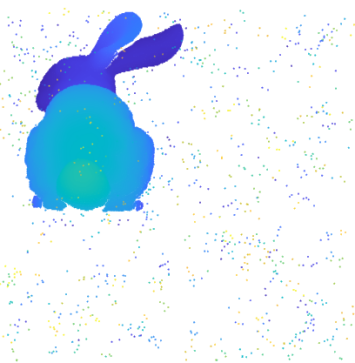

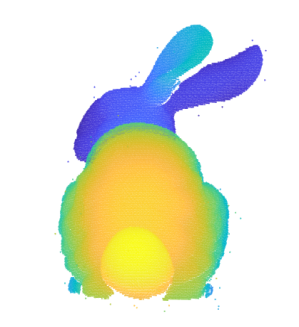


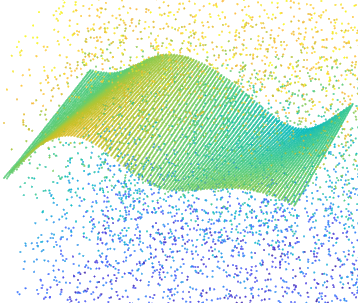

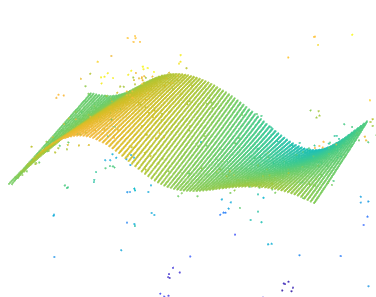

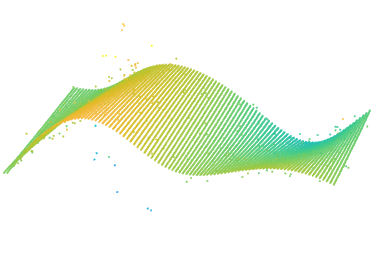


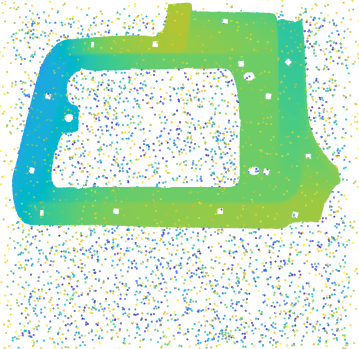

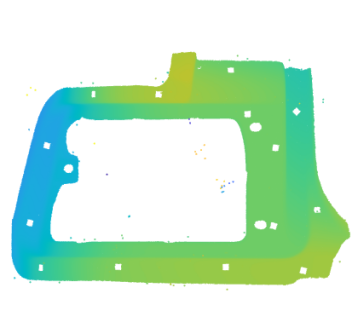

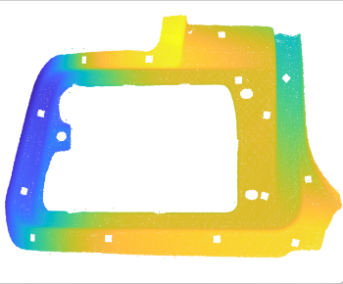


Fig 2


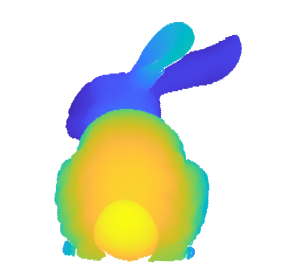


Fig 3


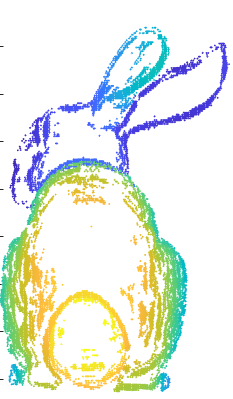


Fig 4


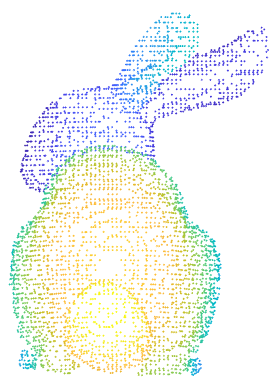


Fig 5


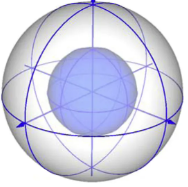


Fig 6

Fig 7


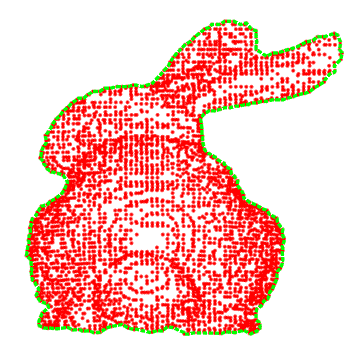


Fig 8


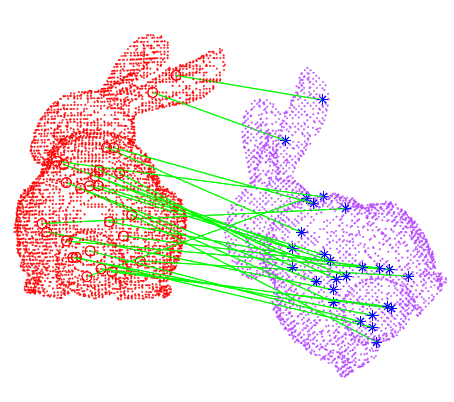


Fig 9


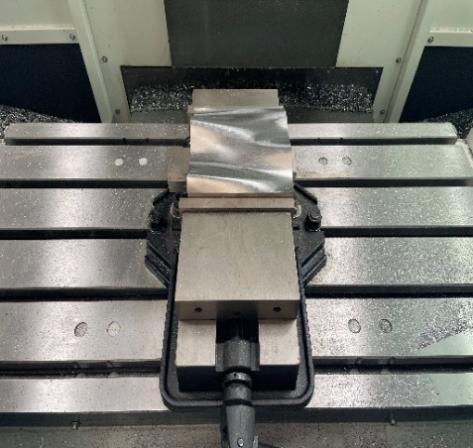


Fig 10


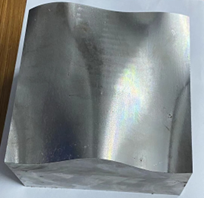


Fig 11


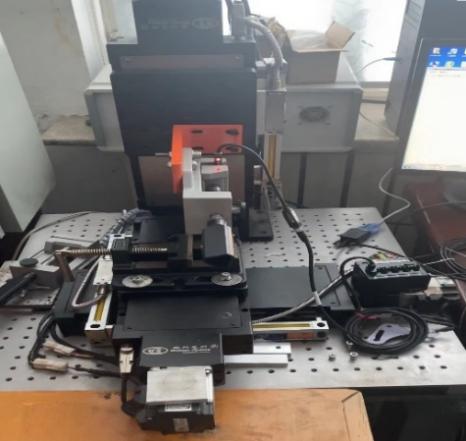


Fig 12


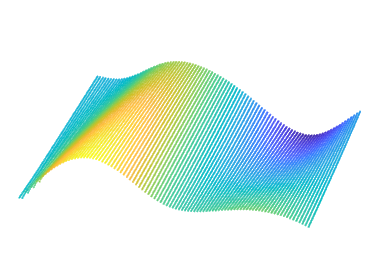


Fig 13


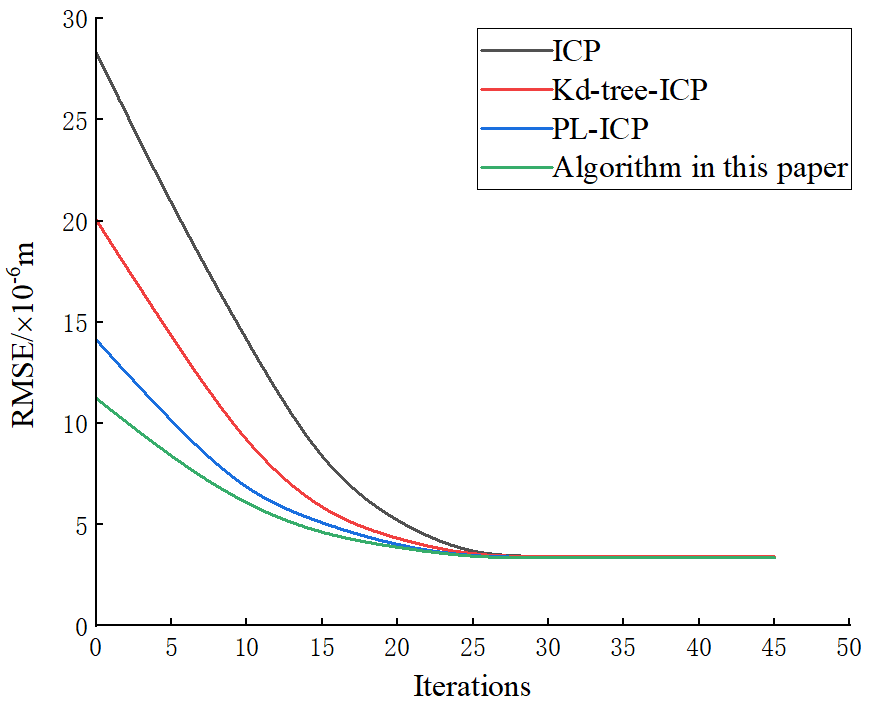

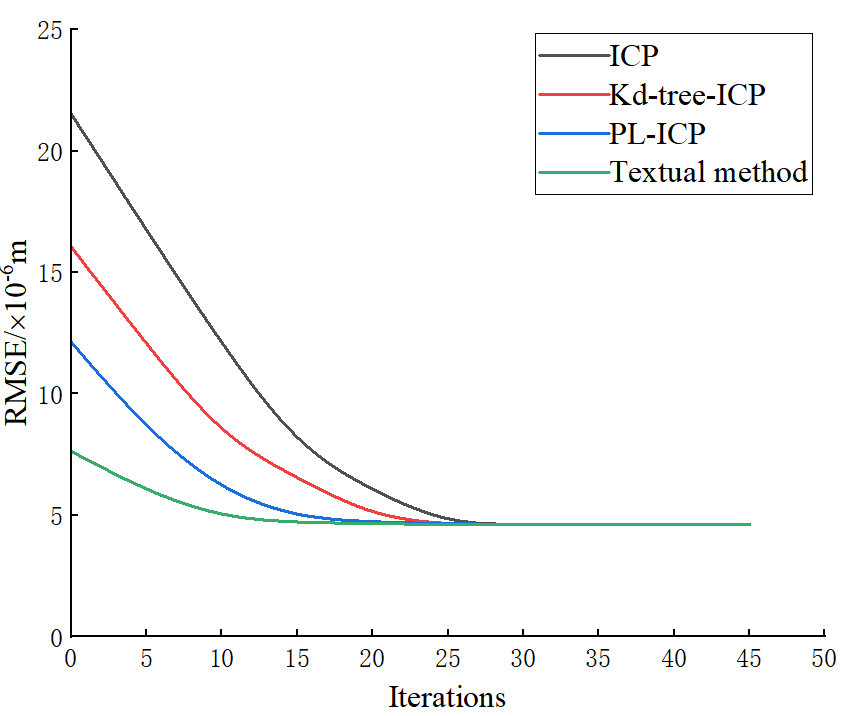


Fig 14

Supplement: S1 Data — (ZIP) [file pone.0296704.s001.zip › Fig.docx]
