# Supplementary material for: Research on 3D point cloud alignment algorithm based on SHOT features
Source: PLoS One. 2024 Mar 27;19(3):e0296704. doi: 10.1371/journal.pone.0296704 (PMC10971591; doi:10.1371/journal.pone.0296704)
Supplement: Attachment — Submitted filename: Response letter.docx [file pone.0296704.s002.docx]

Response letter

Response to reviewer

Dear Editors and Reviewers:

We gratefully thank the editor and all reviewers for their time spend making their constructive remarks and useful suggestions, which has significantly raised the quality of the manuscript and has enable us to improve the manuscript. Each suggested revision and comment, brought forward by the reviewers was accurately incorporated and considered. Below the comments of the reviewers are response point by point-and the revisions are indicated.

Responds to the reviewer’s comments:

Reviewer 1:

1. In the abstract, on line 3, the acronym should be placed in parentheses as follows: "… method based on the Signature of Histogram of Orientation (SHOT) feature is proposed …."

The author's answer : ……directional histogram features (SHOT).

Reviewer 2：

2. In the abstract, on line 9, the mention of "directional histogram features (SHOT)" is confusing. Revising it as "…on normal vector and SHOT features is

proposed…" might be better.

The author's answer: We propose a point cloud registration method based on normal vector and directional histogram features (SHOT).

Reviewer 3:

3. On page 12, please add a reference or web link for the Stanford Bunny dataset.

The author's answer: In this paper, the Stanford Bunny point cloud dataset (https: //www. cc. gatech. edu/ projects/ large_models/ bunny. html) and the self-measured free-form dataset are used for point cloud filtering verification.

I confirm that Figures 1, 2, 3, 4, 5, 6,7,8,9,10,11,12,13 and Table 1 、Table2 are cited in this article.
